# Long-term cerebrovascular effects of cyclophosphamide and vincristine: endothelial senescence, impaired DNA repair signaling, and blood–brain barrier dysfunction

**DOI:** 10.1007/s11357-026-02270-3

**Published:** 2026-04-30

**Authors:** Dorina Nagy, Kiana Vali Kordestan, Roland Patai, Rafal Gulej, Siva Sai Chandragiri, Raghavendra Y. Nagaraja, Santny Shanmugarama, Shoba Ekambaram, Evelyn Brunner, Rebeka Kristof, Mark Nagykaldi, Parikshat Sirpal, Andriy Yabluchanskiy, Karl E. Balsara, Rene Y. McNall, Zoltan Ungvari, Anna Csiszar

**Affiliations:** 1 Vascular Cognitive Impairment, Neurodegeneration and Healthy Brain Aging Program, Department of Neurosurgery, University of Oklahoma Health Sciences Center, Oklahoma City, OK USA; 2 Oklahoma Center for Geroscience and Healthy Brain Aging, University of Oklahoma Health Sciences Center, Oklahoma City, OK USA; 3 Cerebrovascular and Neurocognitive Diseases Research Group, HUN-REN-SU, Semmelweis University, Budapest, Hungary; 4 Doctoral College, Health Sciences Division/Institute of Preventive Medicine and Public Health, International Training Program in Geroscience, Semmelweis University, Budapest, Hungary; 5 Institute of Clinical Pathophysiology, Semmelweis University, Budapest, Hungary; 6 The Peggy and Charles Stephenson Cancer Center, University of Oklahoma Health Sciences Center, Oklahoma City, OK USA; 7 Department of Pediatrics, University of Oklahoma Health Sciences Center, Oklahoma City, OK USA

**Keywords:** Chemotherapy-related cognitive impairment, Endothelial senescence, Vascular aging, Blood–brain barrier permeability, DNA damage

## Abstract

Chemotherapy-related cognitive impairment is increasingly recognized as a long-term consequence of cancer treatment, yet the contribution of the cerebrovascular system remains poorly defined. In this study, we investigated the long-term effects of clinically relevant treatment regimens with cyclophosphamide (CP) and vincristine (VIN) on cerebrovascular cellular senescence, associated molecular signatures, and downstream functional outcomes, including disruption of blood–brain barrier (BBB) integrity in a mouse model. Our results showed that CP induces a persistent cerebrovascular endothelial phenotype characterized by increased cellular senescence, upregulation of mRNA expression of DNA damage checkpoint regulators, and concomitant downregulation of key DNA repair genes. BBB integrity was preserved after CP treatment for larger molecular tracers (40 kDa and 3 kDa) but exhibited increased permeability to small tracers (0.3 kDa), measured by in vivo two-photon microscopy, indicating a subtle yet persistent disruption of barrier function. In contrast, VIN elicited a markedly attenuated and heterogeneous transcriptional response and did not produce detectable BBB impairment, underscoring agent-specific cerebrovascular effects of chemotherapy. Collectively, these findings suggest that chemotherapy induces a persistent cerebrovascular injury phenotype with features resembling vascular aging and reduced vascular resilience, providing a potential mechanistic link between systemic cancer therapy and long-term adverse effects on brain health.

**Supplementary Information:**

The online version contains supplementary material available at https://doi.org/10.1007/s11357-026-02270-3.

## Introduction

Chemotherapy-related cognitive impairment (CRCI) represents a significant and often debilitating consequence of cancer treatment in adult survivors [1], characterized by persistent deficits in memory, attention, and executive function that substantially impair quality of life [2–10]. Accumulating evidence indicates that cerebromicrovascular changes are among the key pathogenic contributors to CRCI [1, 11–16]. Cytotoxic agents are known to induce DNA damage in vascular cells, including endothelial cells, which is a well-established trigger of cellular senescence [17–19]. Senescent endothelial cells exhibit marked morphological and functional alterations and secrete a broad spectrum of senescence-associated factors [20–22], promoting inflammation, vascular dysfunction, and barrier disruption. Notably, these features closely resemble those observed during chronological cerebrovascular aging [12, 23], supporting the concept that cancer therapy may accelerate vascular aging processes within the brain [1]. This framework provides a mechanistic link between systemic chemotherapy exposure and the increased long-term risk of cerebrovascular complications and cognitive decline observed in cancer survivors [1]. Importantly, these changes may also reflect a reduction in cerebrovascular resilience, defined as the ability of the vascular system to maintain homeostasis and adapt to physiological or pathological stressors.

Although many cytotoxic agents exhibit limited permeability across the blood–brain barrier (BBB) [24–26], endothelial cells within the cerebral microvasculature are directly exposed to these circulating agents. Consistent with this, previous rodent studies demonstrated that chemotherapeutic agents such as paclitaxel, cisplatin, and methotrexate induce endothelial senescence in the brain, leading to pathological outcomes like BBB disruption, microvascular rarefaction, altered cerebral blood flow regulation, and impaired neurovascular coupling [12−16]. These vascular alterations may ultimately contribute to CRCI and increase the long-term risk of cerebrovascular diseases [1].

Cyclophosphamide (CP) and vincristine (VIN) are key components of widely used chemotherapy regimens that have been associated with CRCI in both pediatric and adult cancer survivors [27, 28]. Clinical follow-up studies in patients with medulloblastoma, breast cancer, lymphoma, or leukemia receiving CP or VIN-containing regimens have reported persistent cognitive deficits, as well as late-onset vascular complications that may contribute to neurocognitive dysfunction [9, 28–35]. Although these outcomes are usually attributed to multi-agent exposure, the distinct mechanisms of action and toxicity profiles of CP and VIN raise the possibility that each agent may independently contribute to long-term cerebrovascular and neurological injury.

Mechanistically, CP and VIN exert different, but potentially convergent forms of cellular stress that may ultimately promote vascular injury. CP, an alkylating agent, is metabolized into phosphamide mustard which induces DNA cross-linking and DNA damage-signaling, a process known to promote cellular senescence [36, 37]. VIN, an antimitotic agent, disrupts microtubule polymerization and mitotic spindle formation, leading to cell-cycle arrest and cytoskeletal dysfunction [31, 38]. Beyond their antitumor effects, both agents have been implicated in neurotoxic and vascular adverse effects, including white matter injury, and microvascular alterations [35, 39–42], suggesting potential vulnerability of the neurovascular unit.

**Fig. 1:**
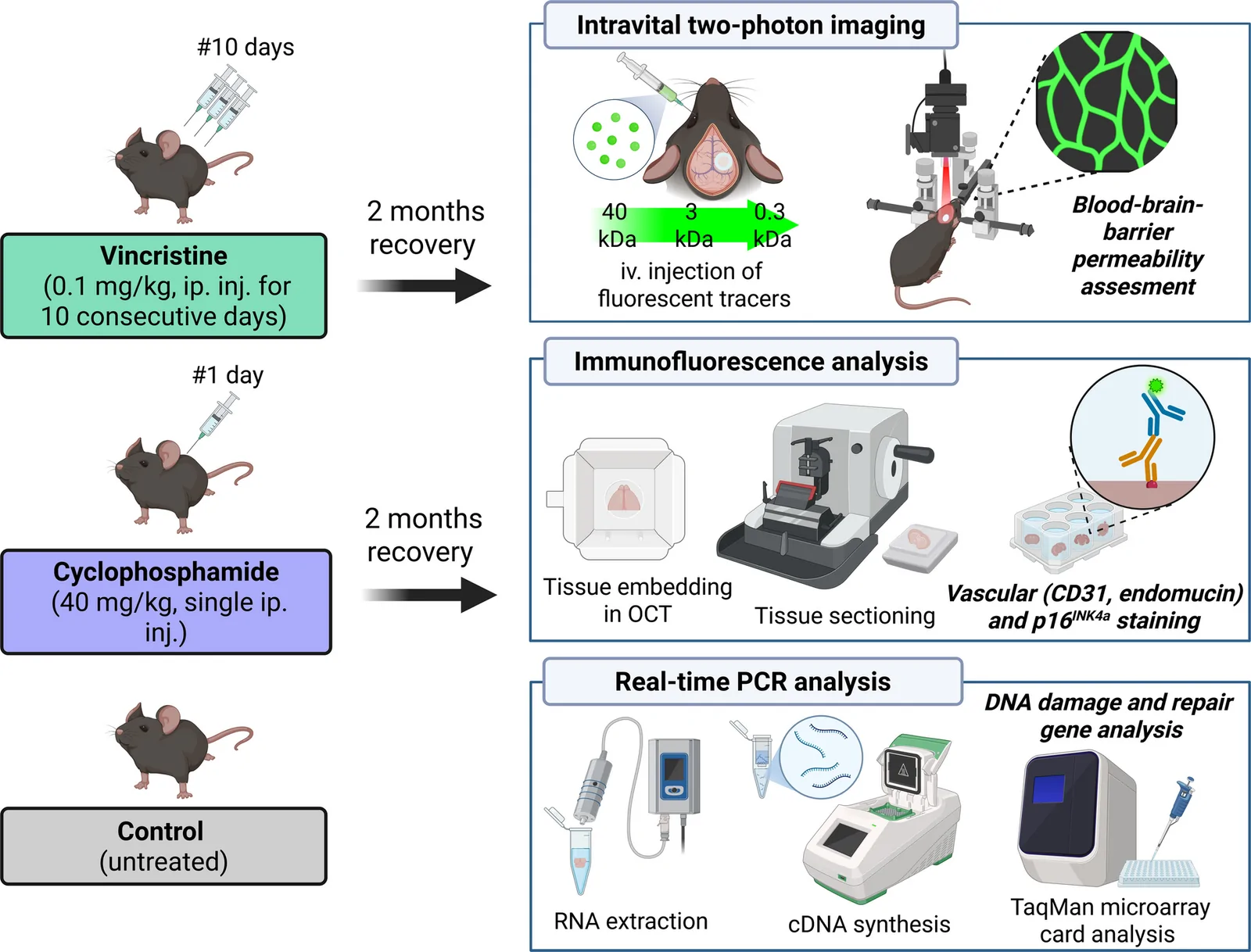
Experimental design. The study compares two chemotherapy-treated groups (vincristine and cyclophosphamide) with age-matched untreated control mice. Following completion of chemotherapy and a 2-month recovery period, outcomes were assessed by intravital two-photon imaging of blood–brain barrier permeability, immunohistochemical analysis of cellular senescence in brain sections, and transcriptional profiling of DNA damage and repair pathways using quantitative RT-PCR

Despite recent preclinical advances highlighting the vascular contribution to CRCI pathogenesis [12−16], the underlying mechanisms are not entirely understood, and the degree to which individual chemotherapeutic agents exert endothelial damage is not fully established. The objective of this study was to further characterize the effects of commonly used chemotherapeutic drugs on endothelial senescence, associated molecular markers, and downstream consequences such as BBB disruption. Therefore, we examined whether a clinically relevant treatment regimen of CP or VIN elicits comparable vascular alterations resembling features of the cerebrovascular aging phenotype (Fig. 1).

## Methods

### Experimental animals

Adult C57BL/6 mice (3–5-month-old, males (*n* = 22) and females (*n* = 22)) were used in this study. All animals were housed in a specific pathogen-free facility at The University of Oklahoma Health Sciences Center (OUHSC) under standard conditions, including a 12-h light/dark cycle and ad libitum access to standard rodent chow and water. To ensure environmental uniformity throughout the experiment, animals were relocated to a conventional animal facility 1 week before initiating chemotherapy treatments.

This study was carried out in full compliance with the principles set forth in the Guide for the Care and Use of Laboratory Animals issued by the National Institutes of Health. All procedures were conducted in accordance with guidelines approved by the Institutional Animal Care and Use Committee (IACUC) of the OUHSC. This ensured that all experiments were performed with the highest standards of animal welfare and ethical responsibility. All experiments are reported in accordance with the Animal Research: Reporting of In Vivo Experiments (ARRIVE) guidelines for reporting preclinical animal studies.

### Treatment protocol

At 3 months of age, mice were randomly assigned to cyclophosphamide (CP)-treated, vincristine (VIN)-treated, and age-matched untreated experimental groups. VIN (vincristine sulfate; Millipore-Sigma, St. Louis, MO, USA) was administered intraperitoneally at a dose of 0.1 mg/kg daily for 10 consecutive days, based on previous studies [43, 44]. CP (Millipore-Sigma) was administered as a single intraperitoneal injection at 40 mg/kg, based on established dosing regimens reported in previous studies [45, 46]. All experiments were performed 2 months after the treatment to examine the long-term consequences of chemotherapeutic drugs on cellular senescence and associated functional consequences (Fig. 1).

### Analysis of immunofluorescence on brain sections

#### Immunolabeling of brain sections

Treated and control mice were anesthetized with isoflurane (3%) and transcardially perfused with ice-cold phosphate-buffered saline (PBS) at a constant pressure of 100–120 mmHg. Brain tissues were fixed in 4% paraformaldehyde (ChemCruz, Dallas, TX, USA) for 24 h, then saturated in 10% and subsequently 30% sucrose. Brains were coronally divided, embedded in Tissue-Tek OCT compound (Sakura Finetek, Torrance, CA, USA), and frozen on dry ice. Coronal halves were sectioned into 30-µm-thick slices using a CM3050S cryostat (Leica Biosystems, Deer Park IL, USA), and free-floating sections were transferred into cryoprotectant solution (glycerol, ethylene glycol, 1 × PBS, and ddH₂O in a 1:1:1:1 ratio). Sections were stored at −20 °C until staining [47].

For immunolabeling, tissue sections were rinsed in 1 × Tris-buffered saline (TBS) with Tween-20 (TBST) twice and then 1 × TBS. Blocking was performed for 3 h in 0.5% Triton X-100 containing 5% bovine serum albumin (BSA), 2.25% glycine, and 10% normal goat serum. After the blocking step, the following primary antibody cocktail was used: rat anti-mouse CD31 (1:75, BD Biosciences, Becton, NJ, USA, Cat. #550274), rat anti-mouse Endomucin (1:75, EMD Millipore Corp, Burlington, MA, USA, Cat. #MAB2624), and rabbit anti-mouse CDKN2A/p16INK4a (1:250, Abcam, Cat. #AB211542). Antibodies were diluted in a 1:5 mixture of blocking buffer and 1 × TBS. Sections were incubated with the primary antibody solution overnight at 4 °C. Afterwards, sections were rinsed on a rocker in 1 × TBST (2 × 5 min) followed by 1 × TBS (5 min), then incubated with secondary antibodies (Goat Anti-Rat Alexa Fluor (AF) 488 (1:500, Invitrogen, Waltham, MA, USA, Cat. #A11006), Goat Anti-Rabbit AF 647 (1:500, Invitrogen, Cat. #A21244)) for 3 h at room temperature, protected from light. Finally, sections were washed again in 1 × TBST and 1 × TBS, then counterstained with DAPI (1:1000 dilution of 5 mg/mL stock in 1 × TBS) for 5 min on a rocker. After a final rinse, sections were mounted on Premium Plain Microscope Slides (Thermo Fisher Scientific, Waltham, MA, USA) and coverslipped using ProLong Gold Antifade Mountant (Invitrogen) [47].

#### Confocal imaging and image analysis of immunolabeled brain sections

Immunolabeled brain sections were imaged using a Stellaris 8 confocal microscope equipped with a tunable white light laser and Power HyD spectral detectors (Leica Microsystems, Wetzlar, Germany). Whole section tile scans were acquired with an HC PL APO 10x/0.40 CS2 dry objective (Leica Microsystems). Images were captured at 1024 × 1024-pixel format with 400 Hz scan speed in unidirectional mode. HyD detectors were configured for 3-channel sequential imaging to minimize crosstalk: DAPI (excitation: λ405 nm; detector range: λ425–720 nm), Alexa Fluor 488 (excitation: λ491 nm; detector range: λ495–660 nm), and Alexa Fluor 647 (excitation: λ653 nm; detector range: λ660–830 nm). Furthermore, a fourth spectrally distinct channel (excitation: λ579 nm; detector range: λ585–650 nm) was also selected to detect autofluorescent signals. Laser power and detector gain were adjusted systematically to avoid under- or overexposure. To enhance the signal-to-noise ratio, line averaging was set to 2× for all channels except AF 647 which used 4× line averaging for p16^INK4a^-positive signal detection. Z-stacks were collected with 2.4-µm z-plane interval and stitched into montage images representing half coronal sections. Afterwards, anatomically matched cortical regions were selected for quantitative image analysis.

Image analysis was performed in FIJI (ImageJ v1.54p platform; National Institutes of Health, USA). Raw confocal z-stacks were converted to hyperstacks, and then, z-planes were merged using the maximum intensity Z-projection. Channels were separated and individually refined to reduce background noise before thresholding of DAPI (blue, nuclei), AF 488 (green; CD31 and endomucin endothelial markers), and AF 647 (red; CDKN2A/p16INK4a marker to detect senescent cells). Thresholds were applied uniformly across all images. Colocalization of the p16^INK4a^-positive signal with the endothelial marker signal was used to assess endothelial senescent burden. Endothelial senescence-associated signal was quantified using an area-based metric calculated as the positive p16^INK4a+^ signal in endothelial markers (CD31^+^/endomucin) normalized to the total CD31^+^ endothelial area within the cortical region.

### Microfluidic quantitative PCR (qPCR)–based transcriptional profiling of DNA damage and repair genes

For transcriptional profiling of DNA damage and repair pathways, half-brain tissue samples were processed for RNA extraction using the RNeasy Mini Kit (Qiagen, Hilden, Germany) on the QIAcube automated platform (Qiagen). RNA quality and concentration were assessed with a NanoDrop UV-Vis Spectrophotometer (Thermo Fisher Scientific), and 1 µg of RNA per sample was used for cDNA synthesis with the High-Capacity RNA-to-cDNA Kit (Thermo Fisher Scientific), following the manufacturer’s protocol on a standard PCR thermal cycler (Bio-Rad, Hercules, CA, USA). The resulting cDNA was then mixed with TaqMan Universal PCR Master Mix (Applied Biosystems, Waltham, MA, USA) and loaded onto TaqMan Array Microfluidic Cards (Mouse DNA Damage and Repair panel; Thermo Fisher Scientific). Quantitative PCR was performed on the QuantStudio 12 K Flex Real-Time PCR System (Applied Biosystems). Cycling conditions consisted of an initial hold at 50 °C, denaturation at 95 °C, and annealing/extension at 60 °C for 40 amplification cycles.

Results from qPCR were analyzed using R (v4.5.2). Cycle threshold (Ct) values were imported as.csv files and processed using a scripted and reproducible workflow. Candidate housekeeping genes were evaluated for expression stability across all samples prior to normalization. Genes exhibiting stable Ct distributions without evidence of systematic group-dependent variation were considered suitable for normalization and retained for downstream analyses. Sample identifiers and experimental condition labels were validated against a sample metadata file. For each sample, ΔCt values were calculated relative to the geometric mean of the selected housekeeping genes (*B2m*, *Polr2a*, *Ywhaz*, and *Hprt*). Differential gene expression was quantified using the ΔΔCt method, with control samples serving as the reference condition. Log2 fold-change values were calculated as −ΔΔCt, such that positive values indicate increased expression relative to control. Differential expression between control and treatment conditions was assessed on ΔCt values using unpaired two-sample *t*-tests performed independently for each gene. Genes were required to have a minimum of two biological replicates per condition to be included in statistical testing. Raw *p*-values were adjusted for multiple testing using the Benjamini–Hochberg false discovery rate correction. For exploratory analysis of global expression patterns, gene-level expression values were arranged into a gene-by-sample matrix and standardized using row-wise z-score transformation. Hierarchical clustering of genes was performed using Euclidean distance and complete linkage. To assess coordinated transcriptional responses, genes were grouped into predefined DNA damage response and repair pathway modules, including damage sensing and signaling, lesion-specific repair, and replication-associated stress and repair. For each module, a module score was calculated as the mean log2 fold-change of all genes within the module for each treatment condition. These module-level summaries were used to compare pathway-level responses across treatments.

### In vivo* blood–brain-barrier permeability measurement*

#### Chronic cranial window surgery

Cranial window implantation was performed as previously described [48]. All surgical procedures were carried out under sterile conditions to minimize the risk of postoperative infection. Animals were maintained under isoflurane gas anesthesia (2%) via inhalation while their head was secured in a stereotaxic frame. The absence of paw and tail reflexes was frequently monitored to ensure adequate anesthetic depth; furthermore, body temperature was continuously checked and maintained at 37 °C using a heating pad. After shaving and disinfecting the scalp, a midline incision was made to expose the skull. Lidocaine (2% saline solution, Millipore-Sigma) was applied topically to the skull surface before performing craniotomy. A circular skull segment (approximately 4 mm in diameter) was thinned over the somatosensory cortex, located 2–3 mm posterior to the coronal suture and lateral to the sagittal suture, using a K.1070 high-speed microdrill (Foredom Electric Company, Bethel, CT, USA) then the skull was carefully removed with a fine forceps. Ice-cold sterile saline was applied on the brain to minimize bleeding, swelling, and thermal damage. A sterile glass coverslip (64–0700, Warner Instruments, MA, USA) was placed over the exposed brain and sealed to the surrounding bone using a cyanoacrylate glue. Then, acrylic cement (Dental Cement, ref: 51459, Stoelting) was used to stabilize the window and reinforce the exposed skull. Animals were monitored postoperatively, and buprenorphine (1 mg/kg, subcutaneous injection; Buprenorphine extended release, ZooPharm, Laramie, WY, USA) was administered for analgesia, while enrofloxacin (10 mg/kg, subcutaneous injection; Enroflox 2.27%, Norbrook, Inc., Lenexa, KS, USA) was given once daily for 4 consecutive days for infection prophylaxis as part of postoperative care. Mice were allowed to recover for 2 weeks before the in vivo two-photon imaging.

#### Intravital two-photon microscopy

BBB permeability was assessed using two-photon imaging. VIN-treated mice implanted with cranial windows were anesthetized with isoflurane gas (2%) and stabilized in a stereotaxic frame under a Stellaris 8 DIVE multiphoton microscope (Leica Microsystems) equipped with an InSight X3 tunable ultrafast multiphoton laser (MKS Spectra-Physics, Milpitas CA, USA) and a water-immersion objective (HC FLUOTAR L 25x/0.95 W VISIR (Leica Microsystems)). The emitted fluorescence was collected using non-descanned detectors (Leica Microsystems) with appropriate filter settings for the fluorophore (excitation: λ920 nm, detector range: λ480–660 nm). Cerebral vasculature was visualized by retro-orbital injection of 500 kDa FITC–dextran (16 mg/kg; Millipore-Sigma). Imaging was performed using 1024 × 1024-pixel format with 600 Hz scan speed at 5 µm z-intervals to record z-planes, with the imaging depth of zero defined at the level of the meningeal vessels. Microvessels were imaged at depths of 0–200 µm using constant laser power (5%) and detector gain (100%). Baseline images acquired immediately following 500 kDa tracer injection served as internal background intensity controls. BBB permeability was subsequently assessed using fluorescent tracers of decreasing molecular weights (40 kDa and 3 kDa FITC–dextrans, 0.3 kDa sodium fluorescein; 8 mg/kg; Thermo Fisher Scientific). Following each retro-orbital injection, 10-min time-lapse z-stacks (1 z-stack per minute) were acquired, resulting in a time-resolved hyperstack for dynamic analysis of tracer extravasation.

CP-treated mice were imaged under comparable experimental conditions using a Fluoview FV1000 two-photon microscope (Olympus, Tokyo, Japan) equipped with a 25× water-immersion objective (XLPLN25XWMP, 1.05 NA), an 800-nm excitation laser, and photomultiplier tube detectors, with fluorescence collected in three spectral ranges (λ420–460, λ495–540, and λ575–630 nm), following a previously described protocol [48].

#### Image analysis for quantification of relative BBB permeability

Image analysis was performed using FIJI (ImageJ, v1.54p platform; National Institutes of Health, USA) as previously detailed [48]. Briefly, the image sequences were compiled into time-series z-stacks and subjected to 3D motion and drift correction to ensure accurate alignment across frames. Vascular images acquired following the 500 kDa tracer injection were segmented to generate a binary vessel mask, which was subtracted from the 3D-corrected maximum intensity projections of subsequent tracer images to exclude intravascular signal. This procedure allowed the selective measurement of extravascular signal intensity of the fluorescent tracers at each time point. Integrated density values of extravascular tracer fluorescence from post-subtraction images were measured and normalized to baseline fluorescence obtained with the 500-kDa FITC–dextran, minimizing variability caused by imaging-related fluctuations. Changes in fluorescence intensity (*I* (a.u.)) relative to baseline (*I*₀ (a.u.)) were plotted as a function of time, beginning with the initial 500 kDa baseline recordings and progressing through tracers of decreasing molecular weight. The area under the curve (AUC) of the fluorescence–time plots, reflecting cumulative tracer extravasation over time, was calculated to quantitatively compare tracer leakage between experimental groups as relative solute permeability (*I*/*I*₀).

### Statistical analysis

Statistical analyses were performed using Prism (v10.0, GraphPad Software Inc., La Jolla, CA, USA). Normality was assessed using the Shapiro–Wilk test. Results are presented as mean ± standard error of the mean (SEM) for normally distributed data or as median with interquartile range (IQR) for non-normally distributed data. Group comparisons were conducted using one-way analysis of variance (ANOVA) or the appropriate nonparametric equivalent (Kruskal–Wallis test with Dunn’s post hoc correction), as well as two-way ANOVA followed by Bonferroni post hoc testing where applicable. A *p*-value < 0.05 was considered statistically significant.

## Results

### Chemotherapy induces endothelial senescence in the brain microvasculature

**Fig. 2:**
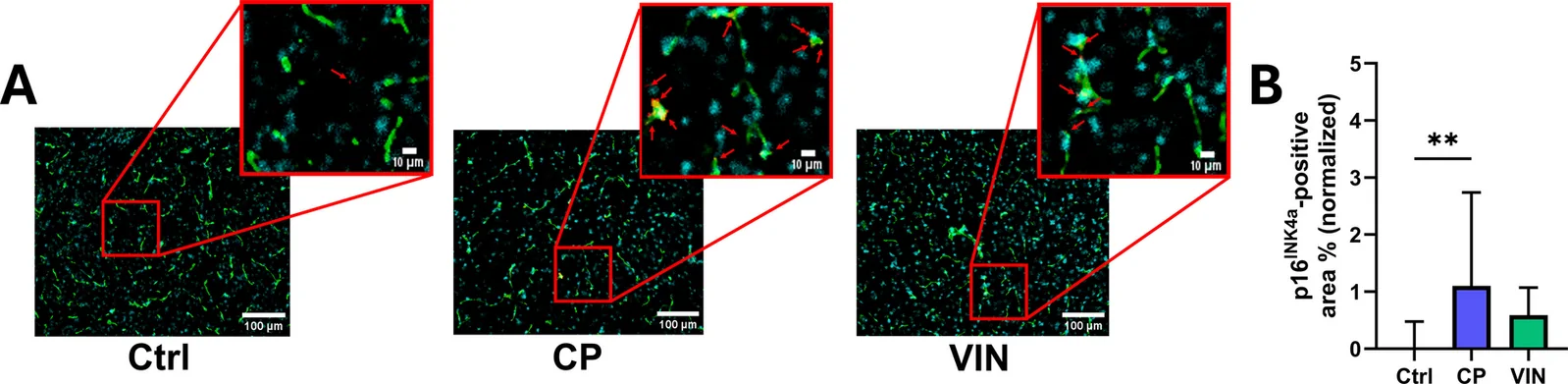
Immunohistochemical assessment of endothelial senescence-associated signal in the brains of mice treated with clinically relevant cyclophosphamide (CP) or vincristine (VIN). **A** Representative confocal images of cortical brain sections from control (Ctrl), CP-treated, and VIN-treated mice stained for endomucin/CD31 (green), p16^INK4a^-positive (red puncta), and DAPI (blue). Insets highlight p16^INK4a^-positive signals overlapping with endothelial marker (arrows), indicating senescence-associated signals within the cortical microvasculature. **B** Quantification of p16^INK4a^-positive endothelial area, expressed as p16^INK4a^^+^/endomucin-CD31^+^ overlapping area normalized to endomucin-CD31^+^ endothelial area, shows a significant increase in CP-treated mice compared with controls, whereas VIN-treated mice did not significantly differ from controls. Data are presented as median with interquartile range (IQR), *n*(Ctrl) = 7, *n*(CP) = 9, *n*(VIN) = 9 brains analyzed; Kruskal–Wallis test with Dunn’s post hoc test, **p < 0.01. Scale bars: 100 µm and 10 µm in the magnified inset

To determine whether chemotherapeutic agents induce senescence within the brain microvasculature, we assessed expression of the senescence marker p16^INK4a^ in cerebral microvessels using immunofluorescence labeling. Brain endothelial cells were identified by co-staining with the endothelial markers endomucin/CD31, allowing specific localization of senescence-associated signals to the vascular endothelium. Representative images show minimal p16^INK4a^ signal in control brains, with only limited endothelial-associated p16^INK4a^-immunreactivity detected (Fig. 2A).

In contrast, chemotherapy exposure markedly increased p16^INK4a^ expression within the brain microvascular network. CP-treated animals exhibited a pronounced increase in p16^INK4a^ signal overlapping with endothelial markers along small vessels and capillaries, consistent with widespread endothelial senescent burden. VIN treatment also increased endothelial-associated p16^INK4a^ immunoreactivity compared with controls, although the magnitude of this effect appeared less robust than that observed following CP exposure.

Quantitative analysis confirmed a significant increase in normalized p16^INK4a^-positive endothelial area in the CP-treated group compared with controls (**p < 0.01; Fig. 2B). In contrast, VIN treatment resulted in a more modest increase that did not reach statistical significance. These findings indicate that chemotherapeutic stress differentially increases senescence burden within the brain microvascular endothelium, with CP exerting a particularly strong pro-senescent effect.

### Chemotherapy induces persistent dysregulation of DNA damage and repair pathways in the brain microvasculature

**Fig. 3:**
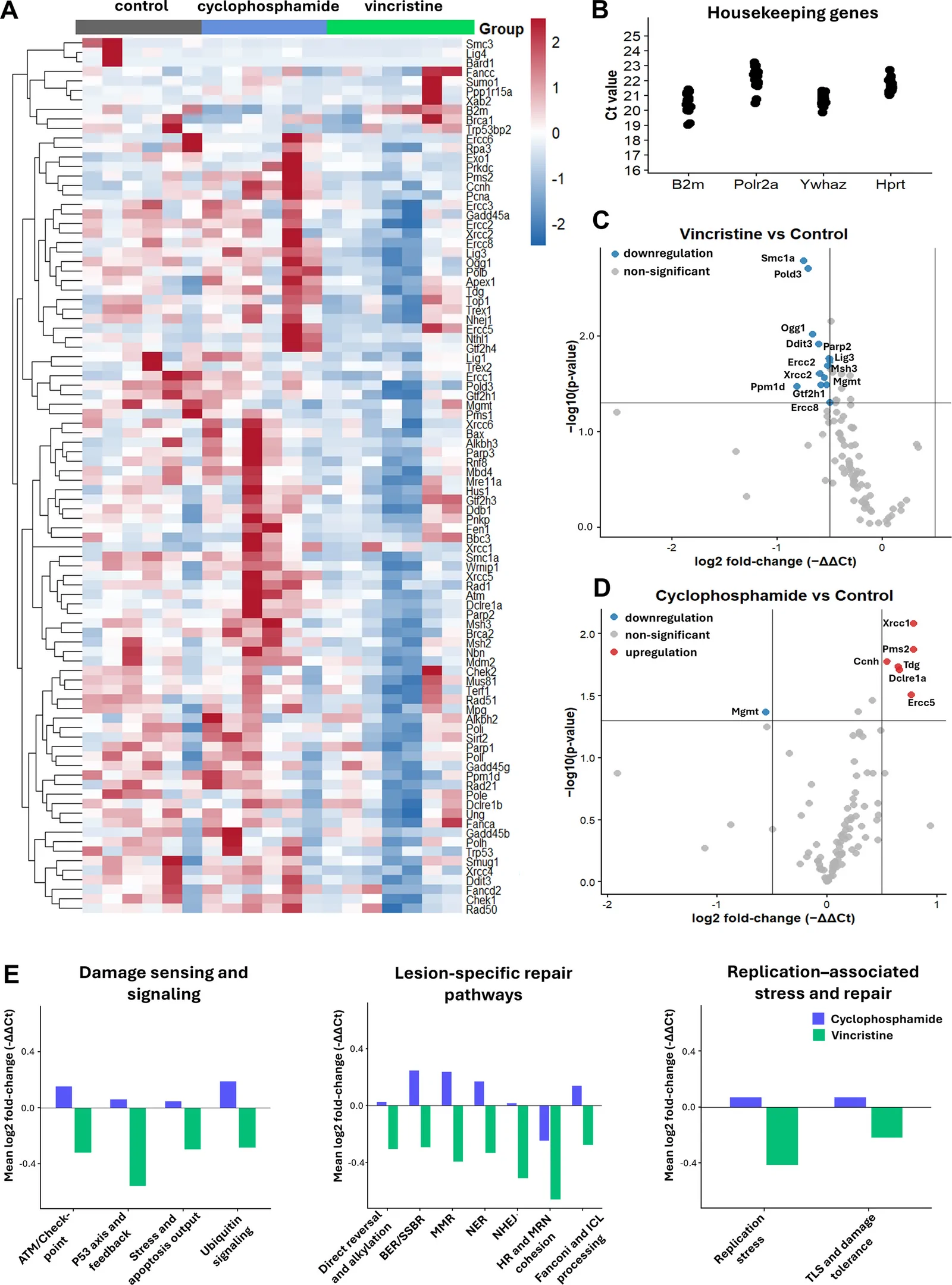
Chemotherapy-induced alterations in DNA damage response and repair gene expression in mouse brain. **A** Heatmap of DNA damage sensing, signaling, and repair gene expression patterns across experimental groups (control, cyclophosphamide, vincristine). Expression values are shown as row-wise z-score–normalized −ΔCt expression across samples, with red indicating relatively higher and blue indicating relatively lower normalized expression for each gene. Hierarchical clustering highlights treatment-specific transcriptional signatures, with cyclophosphamide (CP) producing a distinct expression pattern compared with vincristine (VIN). **B** Ct values of housekeeping genes (B2m, Polr2a, Ywhaz, Hprt) demonstrating stable expression across groups and validating normalization. **C** Volcano plot comparing VIN-treated mice to controls, showing predominantly modest but coordinated downregulation of selected DNA repair genes; blue dots indicate significantly downregulated genes, gray dots indicate non-significant changes. **D** Volcano plot comparing CP-treated mice to controls, revealing both significant upregulation and downregulation of DNA damage response and repair genes, consistent with robust activation of DNA damage signaling pathways. **E** Pathway-level analysis summarizing mean log₂ fold changes (−ΔΔCt) for functional categories, including damage sensing and signaling, lesion-specific repair pathways, and replication-associated stress and repair. CP (blue) preferentially induces damage sensing/signaling and selected repair pathways, whereas VIN (green) is associated with broader suppression of lesion-specific repair and replication stress responses. Data are derived from quantitative PCR-based gene expression analysis of brain tissue; statistical significance thresholds are indicated in volcano plots; *n*(Ctrl) = 7, *n*(CP) = 9, *n*(VIN) = 9 brains analyzed

To characterize transcriptional programs underlying chemotherapy-induced cellular senescence, we profiled expression changes in DNA damage and repair-related genes using a targeted microfluidic PCR array encompassing 96 curated genes involved in DNA damage sensing, repair, checkpoint control, and genomic stability (Supplementary Table 1). Differential expression analysis of DNA damage and repair gene expression patterns revealed a distinct and treatment-specific transcriptional signature across experimental groups (Fig. 3A). CP treatment induced a distinct transcriptional program characterized by coordinated upregulation of DNA damage sensing and signaling genes together with selective suppression of specific repair components, consistent with sustained activation of DNA damage signaling pathways. In contrast, VIN treatment was associated with a more uniform downregulation of genes involved in DNA repair and genome maintenance, without evidence of robust activation of damage-sensing pathways (Fig. 3E). Hierarchical clustering clearly segregated CP-treated samples from both control and VIN groups, indicating agent-specific effects on DNA damage-related transcriptional programs (Fig. 3E).

In VIN-treated mice, a coordinated downregulation of genes involved in multiple DNA repair and genome maintenance pathways was observed. These included components of base excision repair (*Ogg1*, *Lig3*, *Parp2*), nucleotide excision and transcription-coupled repair (*Ercc2*, *Ercc8*, *Gtf2h1*), homologous recombination and chromosomal stability (*Xrcc2*, *Smc1a*), mismatch repair (*Msh3*), and DNA replication and stress signaling (*Pold3*, *Ddit3*, *Ppm1d*) (Fig. 3C). This broad suppression of repair and genome maintenance programs likely reflects reduced replicative and transcriptional demand following VIN-induced mitotic stress, rather than persistent DNA damage signaling. Consistent with this interpretation, VIN treatment did not elicit sustained upregulation of canonical DNA damage checkpoint regulators, suggesting limited engagement of a senescence-associated DNA damage response.

In contrast, CP-treated animals exhibited a broad and largely concordant downregulation of genes involved in DNA repair, accompanied by upregulation of key DNA damage sensing and checkpoint regulators. Specifically, these genes are primarily involved in base excision repair (*Xrcc1*, *Tdg*), mismatch repair (*Pms2*), transcription-coupled and nucleotide excision repair (*Ercc5*), DNA cross-link repair and end-processing (*Dclre1a*), and cell-cycle/DNA damage-linked transcriptional regulation (*Ccnh*), consistent with CP-induced activation of DNA damage response and repair pathways (Fig. 3D). Notably, *Mgmt* downregulation suggests reduced capacity for direct reversal repair of alkylated DNA lesions, consistent with CP’s alkylating mechanism. The selective upregulation of DNA damage sensing and repair genes alongside downregulation of *Mgmt* is consistent with persistent, unrepaired DNA lesions that sustain chronic DNA damage signaling, a hallmark trigger of cellular senescence induction rather than efficient lesion resolution.

These transcriptional changes are consistent with alterations in DNA damage response and repair-related programs but do not directly demonstrate functional impairment of DNA repair capacity.

### CP treatment selectively increases BBB permeability to small molecules

Given our ex vivo evidence that CP treatment induces endothelial senescence in the brain, we next examined whether this cellular phenotype is associated with functional alterations in BBB integrity. BBB permeability was assessed in vivo using intravital two-photon microscopy following systemic administration of fluorescent tracers spanning a range of molecular sizes (40 kDa, 3 kDa, and 0.3 kDa).

**Fig. 4:**
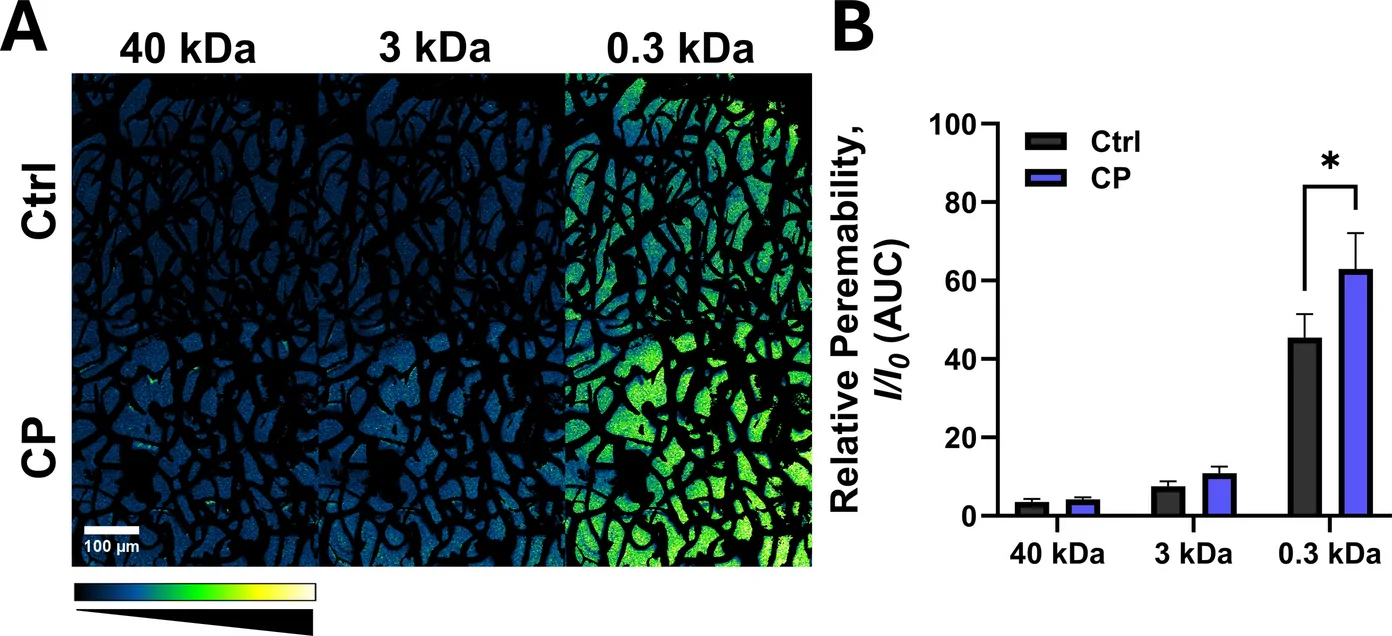
Disruption of blood–brain barrier integrity to small molecules in cyclophosphamide (CP)-treated mice.** A** Representative two-photon microscopy images of cerebral microvasculature in CP-treated and untreated control (Ctrl) mice following retro-orbital injection of FITC–dextran tracers of decreasing molecular size (40 kDa, 3 kDa, and 0.3 kDa), normalized to baseline fluorescence. CP-treated mice exhibit increased extravascular fluorescence of the 0.3 kDa tracer, indicating compromised barrier integrity to small molecules, as evidenced by a color shift from dark blue toward yellow. **B** Quantification of relative permeability expressed as area under the curve (AUC) of normalized fluorescence intensity changes (*I*/*I*₀). CP-treated mice show significantly elevated permeability to the 0.3 kDa tracer compared with controls, whereas no differences are observed for the 40 kDa or 3 kDa tracers. These findings indicate a selective weakening of the barrier to small but not larger molecules. Data are shown as mean ± SEM, *n*(CP) = 5, *n*(Ctrl) = 6; two-way ANOVA with Bonferroni post hoc test, *p < 0.05. Scale bar: 100 µm

Qualitative imaging revealed minimal parenchymal accumulation of the 40 kDa and 3 kDa tracers in both control and CP-treated animals, indicating preserved barrier function to larger molecular weight compounds (Fig. 4A). Consistent with these observations, quantitative analysis of tracer extravasation over time showed no significant differences between groups for either the 40 kDa or 3 kDa tracers (Fig. 4B). In contrast, CP-treated mice exhibited a marked increase in parenchymal accumulation of the 0.3 kDa tracer compared with controls. Quantification of relative permeability, expressed as AUC of tissue fluorescence intensity, confirmed a significant increase in BBB permeability to the smallest tracer in CP-treated animals (*p < 0.05; Fig. 4B). By comparison, VIN-treated mice showed no significant differences in BBB permeability for any of the tracers relative to their corresponding controls (Supplementary Fig. 1).

Together, these data demonstrate that CP induces a sustained and size-selective increase in BBB permeability, characterized by preserved exclusion of larger molecules but enhanced passage of small solutes. This pattern is consistent with a subtle yet persistent disruption of BBB function and suggests that chemotherapy-associated endothelial senescence is accompanied by long-lasting alterations in microvascular barrier properties. In contrast, VIN treatment did not have any significant effect on BBB function, indicating that distinct chemotherapeutic agents differ in their tendency to induce BBB toxicity.

## Discussion

The present study demonstrates that CP induces a persistent cerebrovascular phenotype characterized by endothelial senescence, sustained dysregulation of DNA damage and repair-related transcriptional programs in brain cells, and subtle but functionally significant impairment of BBB integrity. Importantly, these alterations overlap with established hallmarks of vascular aging [21, 49, 50], including genomic instability, cellular senescence, and loss of tissue homeostasis, supporting the interpretation that CP decreases vascular resilience with features resembling cerebrovascular aging. Together, these findings identify the brain microvascular endothelium as a critical and previously underappreciated target of chemotherapy-induced injury and provide mechanistic insight into how systemic cancer therapy may promote long-term neurological vulnerability.

Endothelial senescence is increasingly recognized as a driver of vascular aging and dysfunction [51, 52], contributing to impaired barrier properties, altered inflammatory signaling, and reduced adaptive capacity [23]. Our results extend this concept to cancer therapy, indicating that chemotherapeutic exposure can induce a sustained shift in endothelial state that persists long after treatment. Rather than reflecting an acute toxic effect, these findings support a model in which chemotherapy induces a long-lasting reprogramming of vascular homeostasis [12] that is not readily resolved by endogenous repair mechanisms. Importantly, this phenotype may reflect not only overt dysfunction but also a reduction in vascular resilience, defined as the capacity of the cerebrovascular system to maintain homeostasis and respond to physiological or pathological stressors. In this context, chemotherapy exposure may act as a priming event that shifts the vasculature toward a more vulnerable state, thereby increasing susceptibility to subsequent insults such as aging, inflammation, or metabolic stress.

The transcriptional profiling of DNA damage and repair pathways provides insight into molecular changes associated with this persistent phenotype and demonstrates that CP and VIN engage fundamentally distinct transcriptional programs. CP treatment was associated with coordinated alterations in DNA damage sensing, checkpoint signaling, and repair-related gene expression, whereas VIN induced a more modest and broadly suppressive transcriptional response. These observations warrant further investigation using higher-resolution approaches, such as single-cell or endothelial-enriched transcriptomic profiling, to more precisely define cell-type–specific responses and to delineate the molecular programs underlying chemotherapy-induced neurovascular alterations.

The divergent effects of CP and VIN on endothelial senescence and transcriptional programs likely reflect fundamental differences in their mechanisms of action. CP, an alkylating agent, induces DNA cross-linking and persistent genotoxic stress, which is a well-established trigger of cellular senescence. In contrast, VIN disrupts microtubule dynamics and mitotic spindle formation, leading primarily to mitotic arrest rather than sustained DNA damage signaling. This mechanistic distinction may be particularly relevant in the context of largely quiescent cerebrovascular endothelial cells, which may be more susceptible to genotoxic stress than to mitotic disruption.

However, it is important to note that these conclusions are based on mRNA expression data obtained at a late point (2 months after treatment) and therefore do not directly demonstrate ongoing DNA damage signaling or functional impairment of DNA repair capacity. Instead, these findings are more appropriately interpreted as evidence of long-term alterations in DNA damage/repair-related transcriptional programs following chemotherapy exposure. In addition, transcriptional analyses were performed on whole-brain homogenates, and therefore, the observed gene expression patterns likely reflect contributions from multiple cell types, including neurons, glia, and vascular cells. While endothelial involvement is supported by immunohistochemical evidence of senescence, cell-type–specific attribution of transcriptional changes cannot be definitively established in the present study. Despite these limitations, the persistence of DNA damage/repair-related transcriptional alterations months after chemotherapy exposure may be biologically meaningful. One possibility is that chemotherapy induces a durable “vascular vulnerability” state characterized by reduced genome maintenance reserve or impaired adaptive capacity.

Functionally, CP-induced endothelial senescence and DNA repair dysregulation were accompanied by selective BBB impairment. Using intravital two-photon microscopy with fluorescent tracers spanning different molecular sizes (40 kDa, 3 kDa, and 0.3 kDa), we observed preserved exclusion of larger tracers but significantly increased permeability to the smallest tracer. In line with its comparatively modest transcriptional impact, VIN treatment did not result in a detectable BBB permeability increase, indicating preservation of cerebrovascular barrier function. Fluorescent dextrans and small molecular tracers are widely used to detect mild, size-dependent BBB disruption in vivo [48]. This size-selective “leaky but not ruptured” BBB phenotype is characteristic of early or subtle barrier impairment and is consistent with models in which endothelial aging/senescence promotes BBB permeability changes without gross vascular breakdown [23]. Moreover, BBB dysfunction in aging has been linked to phenotypic shifts in brain endothelial cells and is increasingly viewed as a consequence of endothelial senescence accumulation [51, 53]. Importantly, even modest increases in permeability to small circulating factors, including cytokines, metabolites, and signaling molecules, may have significant effects on the neural microenvironment.

While the association between endothelial senescence and BBB dysfunction is compelling, the present study does not establish a direct causal relationship. However, prior studies using senolytic interventions or genetic clearance of senescent cells provide evidence that endothelial senescence can drive cerebrovascular dysfunction and BBB impairment [12, 51, 54], supporting the proposed mechanism. In line with this, previous studies have shown that chemotherapeutic agents (e.g., paclitaxel, cisplatin, methotrexate) induce endothelial senescence and subsequent BBB dysfunction [12, 14–16], and notably, paclitaxel-induced BBB disruption has been shown to be ameliorated by senolytic treatment [12]. Therefore, future studies will be important to determine whether elimination of senescent cells following CP treatment can similarly prevent or reverse BBB dysfunction, further advancing therapeutic implications.

**Fig. 5:**
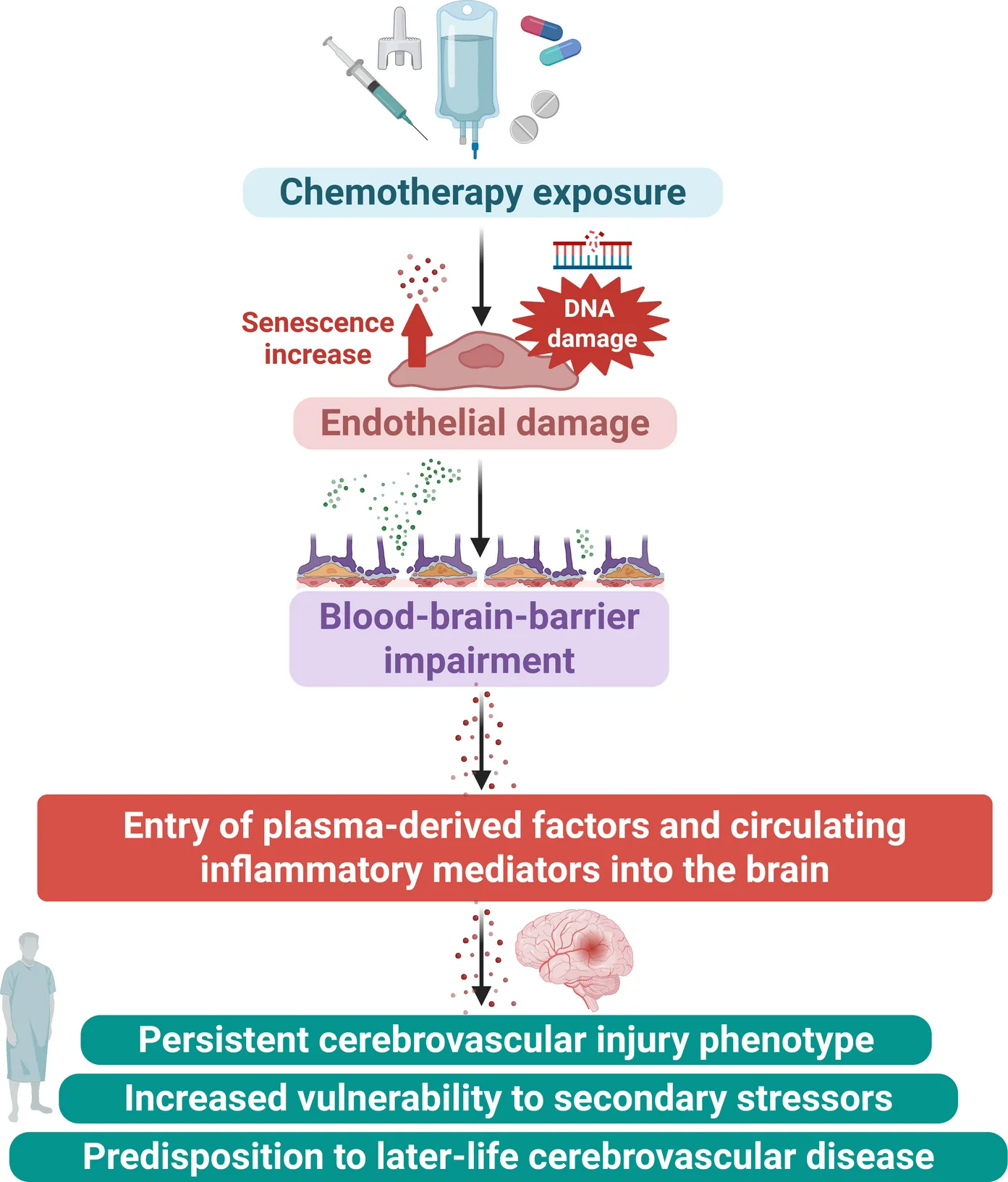
Mechanistic framework linking chemotherapy exposure to persistent cerebrovascular injury and late-life vulnerability. Chemotherapy exposure induces endothelial DNA damage and promotes endothelial senescence, leading to sustained endothelial dysfunction. These alterations drive blood–brain barrier impairment characterized by increased permeability and disrupted vascular integrity. Together, these changes reduce vascular resilience. Consequently, the cerebrovasculature becomes more susceptible to secondary stressors, ultimately increasing the risk of late-onset cerebrovascular disease in cancer survivors

Taken together, our findings support a model in which chemotherapy induces a state of cerebrovascular senescence, including within the endothelium, characterized by persistent DNA damage signaling, impaired repair capacity, and reduced barrier integrity. While endothelial cells remain viable and overall vascular architecture is preserved, the vasculature enters a state of diminished resilience, functionally intact at baseline but less capable of maintaining homeostasis or responding to additional stressors (Fig. 5.). This framework is consistent with the view that BBB dysfunction in aging and disease can arise from gradual endothelial phenotypic drift (including senescence) rather than overt structural failure [23].

Several limitations should be noted when interpreting the present findings. Both male and female animals were included; however, the study was not specifically powered to detect sex-dependent effects. Endothelial senescence was assessed using p16 immunohistochemistry. While it is a widely used marker of senescence-associated burden, additional complementary markers may further refine characterization of the senescent phenotype in future studies. Quantification was performed using an area-based approach, which captures both the extent and intensity of senescence-associated signal, although it does not directly reflect absolute cell number. Transcriptional analyses were conducted on whole-brain homogenates using a targeted gene panel. As such, the observed expression patterns likely reflect contributions from multiple cell types. While endothelial involvement is supported by histological findings, future studies employing cell-type–specific or single-cell approaches will be important to further resolve endothelial-specific transcriptional programs. BBB permeability was evaluated using fluorescent tracers spanning a range of molecular sizes, providing sensitive detection of subtle, size-selective barrier alterations. Future work incorporating endogenous circulating markers may further extend these observations. Finally, the present study focuses on mechanistic cerebrovascular endpoints and does not include behavioral assessments, which in the future will be important to determine how these microvascular alterations relate to neuroinflammation, neurovascular coupling, and cognitive outcomes, as well as to assess their potential reversibility using senescence-targeted or vasoprotective interventions.

In summary, this study demonstrates that cyclophosphamide induces a persistent cerebrovascular endothelial phenotype characterized by endothelial senescence, long-term transcriptional alterations, and selective BBB dysfunction. These findings support a model of reduced vascular resilience as a long-term consequence of systemic chemotherapy with CP.

## Supplementary Information

Below is the link to the electronic supplementary material.

ESM 1 (DOCX.25.5 KB)

ESM 2 Supplementary Material 2 (PNG 269 KB)

High Resolution Image (TIF 952 KB)

## Data Availability

The datasets recorded and analyzed in the current study are not publicly available, but they are available from the corresponding authors upon request.
