# Supplementary material for: Long-term cerebrovascular effects of cyclophosphamide and vincristine: endothelial senescence, impaired DNA repair signaling, and blood–brain barrier dysfunction
Source: GeroScience. 2026 Apr 30;48(5):7407–22. doi: 10.1007/s11357-026-02270-3 (PMC13601448; doi:10.1007/s11357-026-02270-3)
Supplement: Supplementary file 1 [file 11357_2026_2270_MOESM1_ESM.docx]

| **Gene symbol** | **Gene name** | **TaqMan assay ID** | **RefSeq accession** |
| --- | --- | --- | --- |
| 18S | ribosomal RNA | Hs99999901_s1 | X03205.1 |
| Alkbh2 | alkB, alkylation repair homolog 2 (E. coli) | Mm00723434_m1 | NM_175016.2 |
| Alkbh3 | alkB, alkylation repair homolog 3 (E. coli) | Mm01335032_m1 | NM_026944.1 |
| Apex1 | Apurinic/apyrimidinic endonuclease 1 (multifunctional DNA repair enzyme) | Mm01319526_g1 | NM_009687.2 |
| Atm | ataxia telangiectasia mutated homolog (human) | Mm01177457_m1 | NM_007499.2 |
| B2m | beta-2 microglobulin | Mm00437762_m1 | NM_009735.3 |
| Bard1 | BRCA1 associated RING domain 1 | Mm00500274_m1 | NM_007525.3 |
| Bax | Bcl2-associated X protein | Mm00432051_m1 | NM_007527.3 |
| Bbc3 | Bcl-2 binding component 3 | Mm00519268_m1 | NM_133234.2 |
| Brca1 | breast cancer 1 | Mm01249840_m1 | NM_009764.3 |
| Brca2 | breast cancer 2 | Mm01218747_m1 | NM_001081001.1 |
| Ccnh | cyclin H | Mm00445740_m1 | NM_023243.5 |
| Chek1 | checkpoint kinase 1 | Mm01176757_m1 | NM_007691.5 |
| Chek2 | checkpoint kinase 2 | Mm00443844_m1 | NM_016681.3 |
| Dclre1a | DNA cross-link repair 1A | Mm00457569_m1 | NM_018831.4 |
| Dclre1b | DNA cross-link repair 1B | Mm00505657_m1 | NM_133865.2 |
| Ddb1 | damage-specific DNA binding protein 1, 127kDa | Mm00497159_m1 | NM_015735.1 |
| Ddit3 | DNA-damage inducible transcript 3 | Mm01135937_g1 | NM_007837.3 |
| Ercc1 | excision repair cross-complementing rodent repair deficiency, complementation group 1 | Mm00468337_m1 | NM_007948.2 |
| Ercc2 | excision repair cross-complementing rodent repair deficiency, complementation group 2 | Mm00514776_m1 | NM_007949.4 |
| Ercc3 | excision repair cross-complementing rodent repair deficiency, complementation group 3 | Mm00504884_m1 | NM_133658.1 |
| Ercc5 | excision repair cross-complementing rodent repair deficiency, complementation group 5 | Mm01256322_m1 | NM_011729.2 |
| Ercc6 | excision repair cross-complementing rodent repair deficiency, complementation group 6 | Mm01221908_m1 | NM_001081221.1 |
| Ercc8 | excision repair cross-complementing rodent repair deficiency, complementation group 8 | Mm00518465_m1 | NM_028042.3 |
| Exo1 | exonuclease 1 | Mm00516302_m1 | NM_012012.4 |
| Fanca | Fanconi anemia, complementation group A | Mm00516836_m1 | NM_016925.3 |
| Fancc | Fanconi anemia, complementation group C | Mm00514846_m1 | NM_007985.2 |
| Fancd2 | Fanconi anemia, complementation group D2 | Mm01184611_m1 | NM_001033244.3 |
| Fen1 | lap structure-specific endonuclease 1 | Mm01700195_m1 | NM_007999.4 |
| Gadd45a | growth arrest and DNA-damage-inducible, alpha | Mm00432802_m1 | NM_007836.1 |
| Gadd45b | growth arrest and DNA-damage-inducible, beta | Mm00435121_g1 | NM_008655.1 |
| Gadd45g | growth arrest and DNA-damage-inducible, gamma | Mm01352550_g1 | NM_011817.2 |
| Gtf2h1 | general transcription factor IIH, polypeptide 1 | Mm00500417_m1 | NM_008186.4 |
| Gtf2h3 | general transcription factor IIH, polypeptide 3 | Mm00619444_m1 | NM_181410.3 |
| Gtf2h4 | general transcription factor II H, polypeptide 4 | Mm04208001_m1 | NM_010364.3 |
| Hprt | hypoxanthine phosphoribosyltransferase | Mm01545399_m1 | NM_013556.2 |
| Hus1 | HUS1 checkpoint homolog (S. pombe) | Mm00476200_m1 | NM_008316.4 |
| Lig1 | ligase I, DNA, ATP-dependent | Mm00495331_m1 | NM_001083188.1 |
| Lig3 | ligase III, DNA, ATP-dependent | Mm00521933_m1 | NM_010716.2 |
| Lig4 | ligase IV, DNA, ATP-dependent | Mm01221720_m1 | NM_176953.3 |
| Mbd4 | methyl-CpG binding domain protein 4 | Mm01184338_m1 | NM_010774.2 |
| Mdm2 | Transformed mouse 3T3 cell double minute 2, p53 binding protein homolog | Mm01233136_m1 | NM_010786.3 |
| Mgmt | O-6-methylguanine-DNA methyltransferase | Mm00485014_m1 | NM_008598.2 |
| Mpg | N-methylpurine-DNA glycosylase | Mm00447872_m1 | NM_181569.2 |
| Mre11a | MRE11 meiotic recombination 11 homolog A (S. cerevisiae) | Mm00450600_m1 | NM_018736.2 |
| Msh2 | mutS homolog 2 (E. coli) | Mm00500563_m1 | NM_008628.2 |
| Msh3 | mutS homolog 3 (E. coli) | Mm00487756_m1 | NM_010829.2 |
| Mus81 | MUS81 endonuclease homolog (S. cerevisiae) | Mm00472059_g1 | NM_027877.3 |
| Nbn | Nibrin | Mm00449854_m1 | NM_013752.3 |
| Nhej1 | nonhomologous end-joining factor 1 | Mm01259071_m1 | NM_029342.4 |
| Nthl1 | nth (endonuclease III)-like 1 (E.coli) | Mm00476559_m1 | NM_008743.2 |
| Ogg1 | 8-oxoguanine DNA glycosylase | Mm00501781_m1 | NM_010957.4 |
| Parp1 | poly (ADP-ribose) polymerase 1 | Mm01321084_m1 | NM_007415.2 |
| Parp2 | poly (ADP-ribose) polymerase 2 | Mm00456462_m1 | NM_009632.2 |
| Parp3 | poly (ADP-ribose) polymerase family, member 3 | Mm00467486_m1 | NM_145619.2 |
| Pcna | proliferating cell nuclear antigen | Mm00448100_g1 | NM_011045.2 |
| Prkdc | protein kinase, DNA activated, catalytic polypeptide | Mm01342967_m1 | NM_153556.1 |
| Pms1 | postmeiotic segregation increased 1 (S. cerevisiae) | Mm01254621_m1 | NM_153556.1 |
| Pms2 | PMS2 postmeiotic segregation increased 2 (S. cerevisiae) | Mm01200871_m1 | NM_008886.2 |
| Pnkp | polynucleotide kinase 3'-phosphatase | Mm01203158_m1 | NM_021549.2 |
| Polb | polymerase (DNA directed), beta; | Mm00448234_m1 | NM_011130.2 |
| Pold3 | polymerase (DNA-directed), delta 3, accessory subunit | Mm00713051_m1 | NM_133692.2 |
| Pole | polymerase (DNA directed), epsilon | Mm00448288_m1 | NM_011132.2 |
| Polh | polymerase (DNA directed), eta | Mm00453168_m1 | NM_030715.3 |
| Poli | polymerase (DNA directed), iota | Mm01262544_m1 | NM_001136090.1 |
| Poll | polymerase (DNA directed), lambda | Mm00451420_m1 | NM_020032.2 |
| Polr2a | polymerase (RNA) II (DNA directed) polypeptide A | Mm00839493_m1 | NM_009089.2 |
| Ppm1d | protein phosphatase, Mg2+/Mn2+ dependent, 1D | Mm00450393_m1 | NM_016910.3 |
| Ppp1r15a | protein phosphatase 1, regulatory subunit 15A | Mm01205601_g1 | NM_008654.2 |
| Rad1 | RAD1 homolog (S. pombe) | Mm00487885_m1 | NM_011232.2 |
| Rad21 | RAD21 homolog (S. pombe) | Mm00485474_m1 | NM_009009.4 |
| Rad50 | RAD50 homolog (S. cerevisiae) | Mm00485504_m1 | NM_009012.2 |
| Rad51 | RAD51 homolog (S. cerevisiae) | Mm00487905_m1 | NM_011234.4 |
| Rnf8 | ring finger protein 8 | Mm00457985_m1 | NM_021419.1 |
| Rpa3 | replication protein A3 | Mm01165241_g1 | NM_026632.4 |
| Sirt2 | sirtuin 2 | Mm01149204_m1 | NM_022432.4 |
| Smc1a | structural maintenance of chromosomes 1A | Mm00490624_m1 | NM_019710.2 |
| Smc3 | structural maintenance of chromosomes 3 | Mm00484015_m1 | NM_007790.3 |
| Smug1 | single-strand-selective monofunctional uracil-DNA glycosylase 1 | Mm00452897_m1 | NM_027885.3 |
| Sumo1 | SMT3 suppressor of mif two 3 homolog 1 (S. cerevisiae) | Mm01609844_g1 | NM_009460.2 |
| Tdg | thymine-DNA glycosylase | Mm02602088_g1 | NM_011561.2 |
| Terf1 | telomeric repeat binding factor (NIMA-interacting) 1 | Mm00436928_m1 | NM_009352.2 |
| Top1 | topoisomerase (DNA) I | Mm00493749_m1 | NM_009408.2 |
| Tp53 | transformation related protein 53 | Mm01731290_g1 | NM_011640.3 |
| Tp53bp2 | transformation related protein 53 binding protein 2 | Mm00557629_m1 | NM_173378.2 |
| Trex1 | three prime repair exonuclease 1 | Mm00810120_s1 | NM_011637.6 |
| Trex2 | three prime repair exonuclease 2 | Mm01964554_u1 | NM_011907.4 |
| Ung | uracil-DNA glycosylase | Mm00449156_m1 | NM_011677.2 |
| Wrnip1 | Werner helicase interacting protein 1 | Mm00504173_m1 | NM_030215.3 |
| Xab2 | XPA binding protein 2 | Mm00470620_m1 | NM_026156.2 |
| Xrcc1 | X-ray repair complementing defective repair in Chinese hamster cells 1 | Mm00494222_m1 | NM_009532.4 |
| Xrcc2 | X-ray repair complementing defective repair in Chinese hamster cells 2 | Mm00445118_m1 | NM_020570.2 |
| Xrcc4 | X-ray repair complementing defective repair in Chinese hamster cells 4 | Mm00459213_m1 | NM_028012.4 |
| Xrcc5 | X-ray repair complementing defective repair in Chinese hamster cells 5 | Mm00550142_m1 | NM_009533.2 |
| Xrcc6 | X-ray repair complementing defective repair in Chinese hamster cells 6 | Mm00487458_m1 | NM_010247.2 |
| Ywhaz | tyrosine 3-monooxygenase/tryptophan 5-monooxygenase activation protein, zeta polypeptide | Mm03950126_s1 | NM_011740.3 |

***Supplementary Table 1.*** Target genes included in the 96-gene TaqMan microfluidic PCR array covering DNA damage response, repair, checkpoint control, and genomic stability pathways, used to assess chemotherapy-induced (cisplatin, vincristine) gene expression changes associated with endothelial senescence.
